# The Conserved Chromatin Remodeler SMARCAD1 Interacts with TFIIIC and Architectural Proteins in Human and Mouse

**DOI:** 10.3390/genes14091793

**Published:** 2023-09-13

**Authors:** Parysatis Sachs, Philipp Bergmaier, Katrin Treutwein, Jacqueline E. Mermoud

**Affiliations:** 1 Institute of Molecular Biology and Tumor Research, Philipps University Marburg, 35043 Marburg, Germany; 2 CMC Development, R&D, Sanofi, 65926 Frankfurt, Germany; 3 Global Development Operations, R&D, Merck Healthcare, 64293 Darmstadt, Germany

**Keywords:** chromatin organization, nucleosome remodeling, TFIIIC, SMARCAD1, RNA polymerase III, chromatin insulator, tRNA gene, mouse embryonic stem cells, architectural protein

## Abstract

In vertebrates, SMARCAD1 participates in transcriptional regulation, heterochromatin maintenance, DNA repair, and replication. The molecular basis underlying its involvement in these processes is not well understood. We identified the RNA polymerase III general transcription factor TFIIIC as an interaction partner of native SMARCAD1 in mouse and human models using endogenous co-immunoprecipitations. TFIIIC has dual functionality, acting as a general transcription factor and as a genome organizer separating chromatin domains. We found that its partnership with SMARCAD1 is conserved across different mammalian cell types, from somatic to pluripotent cells. Using purified proteins, we confirmed that their interaction is direct. A gene expression analysis suggested that SMARCAD1 is dispensable for TFIIIC function as an RNA polymerase III transcription factor in mouse ESCs. The distribution of TFIIIC and SMARCAD1 in the ESC genome is distinct, and unlike in yeast, SMARCAD1 is not enriched at active tRNA genes. Further analysis of SMARCAD1-binding partners in pluripotent and differentiated mammalian cells reveals that SMARCAD1 associates with several factors that have key regulatory roles in chromatin organization, such as cohesin, laminB, and DDX5. Together, our work suggests for the first time that the SMARCAD1 enzyme participates in genome organization in mammalian nuclei through interactions with architectural proteins.

## 1. Introduction

Genome stability and function in eukaryotes is critically dependent on the organization of chromosomes into distinct functional chromatin domains that are dynamically regulated in space and time to implement cell-type-specific gene expression programs [1,2]. Multiple pathways participate in the demarcation of transcribed chromatin domains from silent heterochromatin, including chromatin-based modifications, insulator complexes, higher-order chromatin organization, an interplay between chromatin and the nuclear periphery, as well as chromatin remodeling. ATP-dependent chromatin remodelers exemplify prime regulators of chromatin architecture and gene regulation. They bind to thousands of sites across the genome and utilize ATP hydrolysis to disrupt histone/DNA contacts to slide, eject, or deposit nucleosomes, thereby impacting virtually all chromatin-based processes [3]. Characterized by the presence of an ATPase/DNA-translocase domain, these enzymes can be divided into four major families: SWI/SNF (SWItch/Sucrose-Non-Fermenting); CHD (Chromodomain Helicase DNA-binding); ISWI (Imitation SWItch); and SWR1/INO80 (Swi2/Snf2-Related1/Inositol Requiring 80). Genetic mutations of chromatin remodelers have been implicated in diseases, for instance, cancer initiation or progression, and frequently lead to impairment of embryonic development and pluripotency, but little is known about the underlying mechanistic basis [4,5]. ATP-dependent remodeling enzymes can influence genome function at different levels [3,6,7] on the one hand by affecting local chromatin structure, for instance, nucleosome patterns and the access of transcription factors and chromatin modifying enzymes to the DNA, and on the other hand, it is emerging that they can contribute to higher-order chromatin architecture, for example, through modulating chromatin loops, insulation of domains, and the 3D position of genetic elements, but little is known about this activity [3,6,7].

In the search for determinants of genome organization, a defined group of DNA binding proteins was identified that can promote long-range chromatin interactions. These are generally referred to as architectural proteins and include condensin and lamins [1,7,8,9]. The best-characterized architectural proteins are CCCTC-binding factor (CTCF) and cohesin complex, which promote the formation of loops and insulate chromatin domains [9,10,11]. It is apparent that several ATP-dependent chromatin remodelers co-localize with CTCF sites, pointing to an extensive interplay that has yet to be fully dissected [7]. Phylogenetically, the most conserved chromatin insulator is the transcription factor IIIC (TFIIIC) [12,13]. This multiprotein complex is best known for promoting the synthesis of transfer RNA (tRNA) genes through the recruitment of the RNA polymerase III transcription machinery [14,15,16]. Besides tRNAs, several TFIIIC/polymerase III-expressed genes encode structural RNAs engaged in translation, e.g., the 5S ribosomal RNA (rRNA) or the signal recognition particle 7SL RNA, underscoring the importance of TFIIIC for normal growth. In yeast [17,18], flies [19], *C. elegans* [20], and mammals [21,22], TFIIIC binds to additional sites in the genome but without the remaining RNA polymerase III apparatus, referred to as extra-TFIIIC (ETC) sites. Work in different species has provided compelling evidence that TFIIIC has extra-transcriptional roles in genome organization, paralleling its fundamental role in transcription [12,13,23,24,25,26]. TFIIIC is required for functional insulator activity of a subset of ETC and tRNA sites, in some instances acting together with polymerase III and in other instances without [18,27,28,29,30,31,32]. It has been found that TFIIIC can cooperate with architectural proteins such as cohesin and condensin [19,21,27,33,34] and mediate long-range chromatin looping [35,36].

As less is known about the role of TFIIIC in mammalian genome organization, studies in yeast have led to the formulation of many of the current models. It was shown that TFIIIC-bound loci act as barriers in *S. cerevisae* and *S. pombe*, preventing intermingling of eu- and heterochromatin features [28,29]. In yeast and *C. elegans*, TFIIIC facilitates recruitment of its targets to the nuclear periphery, although this seems not to be sufficient for insulator function in budding yeast [18,20,24,28,37,38]. In *S. pombe*, a chromatin remodeler belonging to the SWR1/INO80 family, Fft3 (fission yeast Fun30), has emerged as a regulator of TFIIIC-bound insulator elements [31,39]. Fft3 co-localizes with the TFIIIC complex at actively transcribed polymerase III sites, such as tRNA genes located at the border between centromeric and pericentric heterochromatin, and preserves their chromatin state [31]. On the other hand, enrichment of Fft3 at ETC sites was not observed. When Fft3 is depleted, euchromatin invades centromeres [31]. Moreover, tRNA genes lose their peripheral positioning, suggesting that Fft3 has a role in anchoring these sites to nuclear structures [39]. Therefore, it was proposed that chromatin remodeling catalyzed by Fft3 is important for both functional and spatial organization of insulators in *S. pombe* [31,39].

Representing one of the most evolutionary conserved of remodeling complexes, Fft3 homologs are found in all eukaryotes [40,41]. This includes Fun30 in *S. cerevisae*, which is similarly enriched at tDNA [42] and SMARCAD1/Etl1 in mammals [40]. SMARCAD1 has been found mutated or mis-regulated in skin diseases accompanied by adermatoglyphia and in skin, breast, and pancreatic cancer [43,44,45,46]. A homozygous mutation of this remodeler in the mouse results in growth retardation, prenatal-perinatal lethality, reduced fertility, and skeletal abnormalities [47]. Expressed throughout development and in virtually all adult tissues, SMARCAD1 levels are particularly high in the central nervous system, oocytes, and embryonic stem cells (ESCs) [48,49,50]. In mESCs, SMARCAD1 functions in transposon and provirus silencing by modulating the local chromatin structure [51,52,53]. Moreover, this remodeler has been linked to anti-microbial defense [54] and in somatic cells has acknowledged roles during DNA damage repair [55,56], in heterochromatin stability, and in epigenetic inheritance [57,58]. Whether SMARCAD1 operates with TFIIIC in vertebrates has not been addressed, limiting our understanding of the wider role of this remodeler in chromosome boundary function and in 3D genome architecture.

Given the strong evolutionary conservation of both TFIIIC and SMARCAD1, we posed the question regarding to what degree the partnership between TFIIIC and Fft3 is conserved in mammals and what its functional significance is. We found that the mammalian counterparts of these proteins do indeed co-purify, both in somatic and pluripotent cells, but there are fundamental differences. Our gene expression analysis shows that SMARCAD1 is not required for the action of TFIIIC as a general RNA polymerase III transcription factor in mESCs. Rather, we established a connection between SMARCAD1 and several architectural proteins known to mediate long-range interactions and postulate that SMARCAD1 remodeling participates in genome organization in mammalian nuclei. We show that TFIIIC directly interacts with SMARCAD1 enzyme, but surprisingly, these proteins do not share the same binding sites in the mESC genome. These findings lead to a new perspective of SMARCAD1 function in TFIIIC regulation, stimulating future research into the relationship between heterochromatin, chromatin remodeling, and genome organization in pluripotent cells.

## 2. Materials and Methods

### 2.1. Cell Culture

Mouse embryonic stem cells (46C, PGK12.1, E14) were grown on gelatin-coated dishes without feeder cells in Dulbecco’s modified Eagle’s medium (DMEM, Invitrogen, Waltham, MA, USA), supplemented with 10% fetal bovine serum (Invitrogen), 1 × non-essential amino acids, 1 mM sodium pyruvate, 2 mM L-glutamine, 50 µM mercaptoethanol, 10^3^ U/mL LIF (ESGRO, Darmstadt, Germany), and 1% penicillin/streptomycin. Differentiation of ESCs was achieved by removing LIF from the medium [51]. HeLa cells were grown as recommended by the American Type Culture Collection, ATCC. All cell lines were routinely tested with MycoAlert Detection (Lonza, Basel, Switzerland) for mycoplasma contamination. Transfections were performed using Lipofecatamine2000 (ThermoFisher Scientific, TFS, Waltham, MA, USA) according to the manufacturer’s instructions.

### 2.2. Plasmids

To perform Glutathione-S-transferase (GST)-pulldown experiments, we generated a human TFIIIC102 GST-fusion protein. TFIIIC102 cDNA was obtained from OriGene (RC208122), amplified by PCR using Q5 high-fidelity DNA polymerase (forward primer 5′-GAGTCA*GGATCC*ATGTCAGGGTTCAGTCCGGAAC, reverse primer 5′ CACGATGCGGCCGCTTACGTTATAGAACAATAGGTATACAAAAGC), cloned into a pGEX-6P-1 vector using BamH1 and Not1 restriction sites and confirmed by DNA sequencing. GST-fusion proteins were expressed in *E. coli* and purified as described in [59]. His-tagged human SMARCAD1 baculovirus expression construct and GST-KAP1 expression constructs and preparation of these recombinant proteins is described in [59]. Three shRNA sequences were used to knockdown Smarcad1 [51]: Exon 7: 5′-GGACTATAGCAGTTGTGAA-3′ in pHYPER and Exon12: 5′-GTATGAGGATTACAATGTA-3′ and 5′-GAAGAGCGTAAGCAAATTA-3′ in the pSUPER backbone. Knockdown efficiency was determined by a combination of Western blotting, indirect immunofluorescence, or quantitative reverse transcription PCR.

### 2.3. Lysate Preparations, Cell Fractionations, Immunopreciptiations, and Western Blots

Nuclear extracts were prepared as previously described [57,59] and precleared with Dynabeads. Nuclear extract containing 150 μg protein was used for each immunoprecipitation using 3 μg of specific antibody or IgG. Antibodies used were IgG rabbit (NA934V; GE Healthcare, Buckinghamshire, UK); SMARCAD1 (A301-592A; Bethyl, Biomol, Hamburg, Germany); TFIIIC63 (A302-242A; Bethyl, Biomol, Hamburg, Germany); TFIIIC90 (PA5-31288; ThermoFisher Scientific, TFS, Waltham, MA, USA). Furthermore, 150 units/ml Benzonase (Novagen, ThermoFisher Scientific, TFS, Waltham, MA, USA) and 0.1 μg/μL EtBr were present throughout the procedure to reduce interactions mediated by nucleic acid. Immune complexes were captured by Protein G Dynabeads (Novex, ThermoFisher Scientific, TFS, Waltham, MA, USA). Dynabeads–Ab–Ag complexes were washed four times in 20 mM HEPES, pH 7.6, 100 mM KCl, 0.2 mM EDTA, 1.5 mM MgCl_2_, 0.5 mM DTT, 20% glycerol, and 0.05% Triton X-100 containing protease inhibitors and then once in the same buffer but with additional 50 mM NaCl. Bound proteins were eluted with SDS sample buffer and analyzed by SDS-PAGE and immunoblotting. Gels were blotted onto PVDF or nitrocellulose membrane and blocked for 1 h in PBS + 0.1% Tween20 containing 5% (*w*/*v*) skimmed milk before an overnight incubation with primary antibody at 4 °C. The membranes were then probed with HRP-conjugated secondary antibodies diluted in PBS + 0.1% Tween20 containing 5% (*w*/*v*) skimmed milk powder. After washing, membranes were developed with an enhanced chemiluminescence detection system (ECL Western blotting Detection Reagent; GE Healthcare, Buckinghamshire, UK) and exposed to X-ray film or developed digitally using the ChemiDoc (Bio-Rad, Hercules, CA, USA) imaging system.

Biochemical fractionation of PGK12.1 *Ctrl*-KD and stable *Smarcad1-*KD ESCs [59] into soluble and chromatin fractions was performed according to [59] using CSK buffer (10 mM PIPES, pH 6.8, 300 mM sucrose, 100 mM NaCl, 3 mM MgCl_2_, 1 mM benzamidine, and 0.5 mM PMSF) and four minutes of extraction with 0.1% Triton X-100. The extract amounts corresponding to the equal numbers of starting cells were analyzed by SDS-PAGE and immunoblotting.

### 2.4. Antibodies

Commercially available antibodies and dilutions used in Western blots include: SMARCAD1 (A302-593A, Bethyl, Biomol, Hamburg, Germany) 1:1000 and (HPA016737, Sigma-Alderich, St. Louis, MO, USA) 1:5000; TFIIIC90 (PA5-31288, ThermoFisher Scientific, TFS, Waltham, MA, USA) 1:500; TFIIIC63 (A301-242A, Bethyl, Biomol, Hamburg, Germany) 1:1000; TFIIIC220 (sc-34542, Santa Cruz, Dallas, TX, USA) 1:200; TFIIIC102 (H00009330-MO1, Novus Biologicals, CO, USA); TFIIIC110 (ab 89113, Abcam, Cambridge, UK) 1:500; SMC1 (A300-055A, Bethyl, Biomol, Hamburg, Germany) 1:2500; SMC3 (ab 9263, Abcam, Cambridge, UK) 1:1000; DDX5 (A300-523A, Bethyl, Biomol, Hamburg, Germany) 1:5000; LaminB1 (ab 16048, Abcam, Cambridge, UK) 1:1000; H3 (06-599, Millipore, Sigma-Alderich, St. Louis, MO, USA) 1:10,000; GAPDH (sc25778, Santa Cruz, Dallas, TX, USA) 1:500.

### 2.5. GST-Pulldown Assay

Packed glutathione Sepharose 4B beads (25 µL) with similar protein loading (13 µg protein) where washed two times in buffer (100 mM NaCl, 20 mM Hepes, 5% Glycerol, 0.1% Triton, 0.2 mM PMSF, and 0.2 mM Benzamidine) and blocked with 1% fish gelatin and 0.1 mg/mL BSA for 1 hour at 4 °C. After three times washing in buffer, the pulldown was performed in a total volume of 150 µL buffer with 1 µg of His-Smarcad1 produced in baculovirus as described in [59]. After incubation of 1.5 h at 4 °C, the beads where washed three times with buffer before the protein was eluted twice: once with 20 µL and then with a further 10 µL 1 × SDS loading dye. Bound proteins were detected by Western blot.

### 2.6. Chromatin Immunoprecipitation

Chromatin immunoprecipitation was performed using the One Day ChIP kit (Diagenode C01010080) following double-cross-linking of ESCs using the protocol provided by the manufacturer with a Biorupter (Diagenode, Seraing, Belgium) as described in [51] but optimized for TFIIIC to 5 minutes of shearing. The following antibodies were used (3 ug): SMARCAD1 (HPA016737, Sigma-Alderich, St. Louis, MO, USA) and TFIIIC63 (A302-242A, Bethyl, Biomol, Hamburg, Germany). ChIP DNA was analyzed in triplicate using real-time PCR with Sybr Green (Bio-Rad, Hercules, CA, USA) on an Agilent MX3000P as described in [51]. Primer sets used for qPCR are available in Supplementary Table S1. qPCR conditions were as follows: 10 min at 95 °C followed by 40 cycles at 95 °C for 15 s and 60 °C for 30 s, followed by a plate read after each cycle. Melting curve test was performed at the end of each experiment (from 55 to 95 °C, plate read every 0.5 °C) to ensure the specificity of amplification.

### 2.7. ChIP-Seq Analysis

The SMARCAD1 ChIP dataset was derived from PGK12.1 ESCs [51] and downloaded from the ArrayExpress repository E-MTAB-7012. TFIIIC binding sites (peaks) were called from published ChIP seq data [21] (ArrayExpress Accession-Number E-MTAB-767) for TFIIIC-220 and TFIIIC-110 (ERR045701) and the corresponding control (ERR045693) following the peak calling protocol described in [51]. Only peaks found in both ChIP-experiments, namely TFIIIC-220 and TFIIIC-100, were kept (common TFIIIC peaks). To obtain the tRNA genes that are bound by TFIIIC, we filtered the lists of tRNA genes retrieved from the ucsc download, table rmsk, filter repclass = ‘tRNA’ down to those tRNA genes that overlap with the common TFIIIC peaks. To compare TFIIIC-binding sites with SMARCAD1-binding sites, common TFIIIC peaks were expanded by 500 bp at their start and stop, respectively. In case these enlarged common TFIIIC peaks overlapped with each other, overlapping peaks were merged.

### 2.8. Gene Expression Analysis

Total RNA was DNAse digested and reverse transcribed with SuperScript II and random hexamers as described in [51]. qPCR reactions were performed using a CFX Connect Real-Time PCR Detection System (Bio-Rad, Hercules, CA, USA) with iTaq Universal SYBR Green (Bio-Rad, Hercules, CA, USA). Pairs of primers were evaluated for generating single-peak melting profiles and for linear amplification over a range of DNA template dilutions. qPCR assays were performed in triplicates and normalized to housekeeping genes (*Gapdh*, *Hsp90ab1*, and *Atp5b*). Expression levels were calculated with the Bio-Rad CFX Manager software (version 3.1), which uses a ΔΔCq calculation method. Primers are listed in Supplementary Table S1. Primers were designed with PrimerBlast using in silico PCR (UCSC browser) after selecting tRNAs reported to be bound by polymerase III and TFIIIC in a genome-wide binding study carried out in 46C ESCs by [21]; ETC sites were chosen from the same publication and are TFIIIC sites not bound by RNA polymerase III subunit RPC4. Each primer pair was tested for efficiency and quantification with serial 10-fold dilutions of genomic DNA.

## 3. Results

### 3.1. Human Remodeler SMARCAD1 Interacts with TFIIIC and Chromatin Architectural Proteins

**Figure 1:**
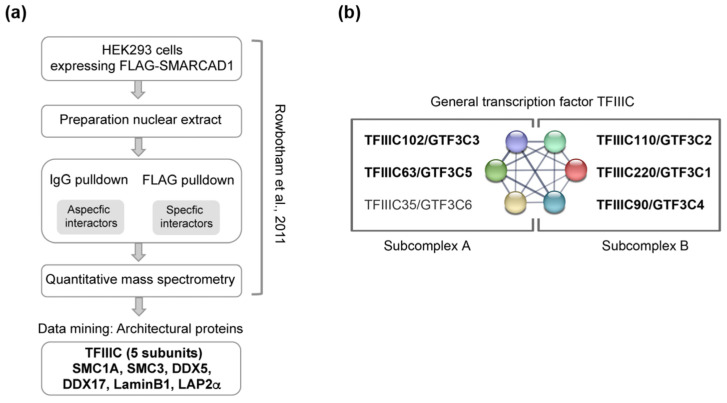
Data mining of FLAG-SMARCAD1 affinity purification from human cells reveals co-purification of architectural proteins. (**a**) Flow chart of the experimental approach used in [57]. IgG and FLAG pulldowns were performed, and peptides were quantified using label-free mass spectrometry. Identified peptides for the proteins we validated here are shown in Supplementary Figure S1. (**b**) The six polypeptides of the hTFIIIC complex and their known interactions according to the STRING physical network. TFIIIC complex division into two sub-complexes is indicated; modules A and B bind A and B box DNA loci, respectively [60]. Shown in bold are the five subunits that were identified by mass spectrometry as associated with tagged, overexpressed SMARCAD1 by [57].

An unbiased proteomics screen performed previously revealed that in human somatic cells, SMARCAD1 interaction partners are predominantly involved in DNA repair, DNA replication, and transcriptional repression [57]. Transcription factors, on the other hand, were not enriched in the SMARCAD1 interactome, with the notable exception of the RNA polymerase III general transcription factor TFIIIC. Peptides corresponding to five of the six members of the TFIIIC complex (TFIIIC220,110,102, 90, and 63) were prominent in the interactome from HEK293 cells (Figure 1; Supplementary Figure S1) [57]. Given the dual role of TFIIIC in transcription and chromosome organization [13,15,16,22,60], a partnership with TFIIIC may reflect a role of SMARCAD1 in the regulation of RNA polymerase III transcription or alternatively in chromosome architecture or possibly a contribution to both. We therefore interrogated the published SMARCAD1 interactome from the HEK293 cell for the presence of other proteins with acknowledged roles in either polymerase III transcription or chromatin architecture alongside TFIIIC. While additional components of the polymerase III transcription machinery were not apparent, we identified cohesion subunits (SMC1A and SMC3), laminB1, lamina-associated polypeptide LAP2Aalpha, and DEAD box RNA helicases (DDX5 and DDX17), all of which have been reported to participate in chromosome organization (Figure 1; Supplementary Figure S1) [21,22,61,62]. For example, lamins are involved in tethering the genome to the nuclear membrane, thereby creating a repressive environment for transcription [63]. Cohesin has been shown to co-localize with TFIIIC in the genomes of yeast, flies, and mammals and is thought to cooperate with TFIIIC in chromatin organization [19,21,22,33,64]. We thus selected SMC1A and SMC3 for further analysis as well as DDX5 (aka p68), which has been linked to insulator function mediated by CTCF [62,65].

**Figure 2:**
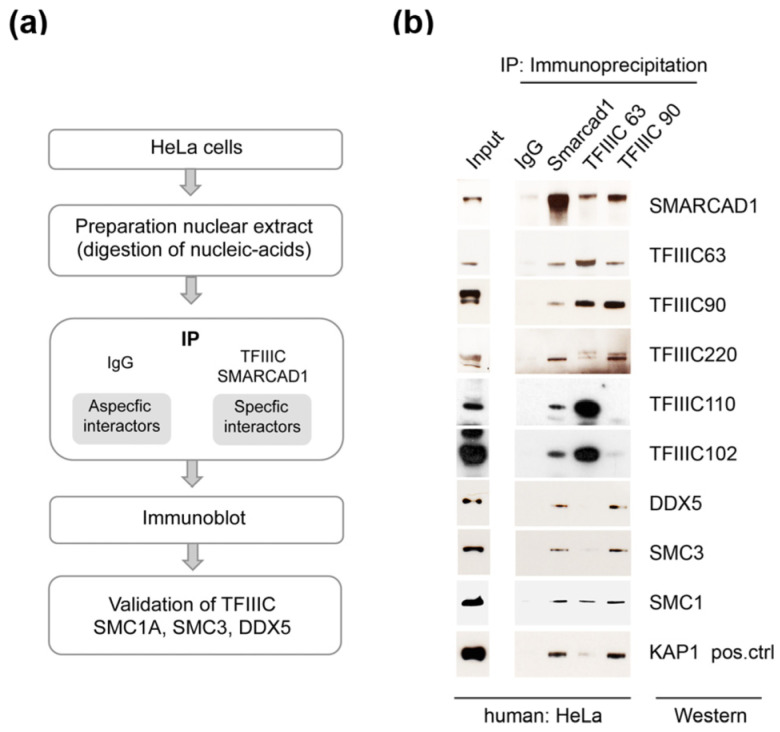
Validation of architectural proteins as bona fide SMARCAD1-associated proteins by reciprocal co-immunoprecipitation of endogenous proteins. (**a**) Flow chart of the experimental approach. (**b**) Endogenous co-immunoprecipitation experiments (n = 2–4) from nuclear extracts from HeLa cells with anti-SMARCAD1 or anti-TFIIIC antibodies (subunits 63 and 90) analyzed by Western blot with the indicated antibodies. Inputs, 9% of the nuclear extract, are from the same blot as the IP. IgG, immunoglobin G. KAP1, a known binding partner of SMARCAD1, was analyzed in parallel (positive control).

The initial identification of these candidate SMARCAD1 interactors was based on immunoprecipitation–mass spectrometry (IP-MS) results obtained using an ectopically overexpressed FLAG-tagged SMARCAD1 as bait for affinity purification (Figure 1a). As overexpression of proteins can affect complex assembly [66], we wanted to ensure that we could detect a specific interaction of TFIIIC and architectural proteins with SMARCAD1 when endogenous proteins at native expression levels were investigated. To this end, we performed co-immunoprecipitation experiments in HeLa nuclear extracts with antibodies recognizing the two distinct TFIIIC sub-complexes, which together form a stable functional complex (Figure 2). Structural, biochemical, and genetic approaches have implicated the largest subunits, namely TFIIIC102 and TFIIIC220, in linking the two sub-complexes [60,67,68,69]. A presentation of known extensive interactions between the individual subunits taken from the STRING database (https://string-db.org accessed on 14 July 2023) is shown in Figure 1b. For example, in humans, it has been shown that TFIIIC102 interacts with TFIIIC63 via a tetratricopeptide repeat [68].

Endogenous SMARCAD1 was efficiently co-immunoprecipitated with antibodies targeting TFIIIC63 or TFIIIC90, components of the A and B complex, respectively, but not with an IgG antibody (Figure 2a,b). Figure 2 shows that the TFIIIC63 antibody not only precipitates SMARCAD1 and components of the A complex such as TFIIIC102 but also components of the B complex, as expected. While the TFIIIC90 antibody also pulls down architectural proteins (DDX5, SMC3, and SMC1), a stable association with TFIIIC102 and 110 was not apparent in HeLa cells. The reasons are not clear but could reflect cell-specific properties of the sub-complexes, for instance, secondary modifications or stability. These experiments were conducted in the presence of benzonase and ethidium bromide, ensuring that observed associations were mediated by protein–protein interactions rather than through nucleic acids. We further verified these associations by reciprocal co-immunoprecipitation experiments with an anti-SMARCAD1 antibody (Figure 2b). KAP1, a known stoichiometric binding partner of SMARCAD1, co-immunoprecipitated with SMARCAD1 in agreement with previous reports [57,59]. In addition, we identified TFIIIC subunits (63, 90, 110, 120, and 220), cohesin subunits (SMC1A and SMC3), and the RNA helicase DDX5 as stably associated with SMARCAD1. Collectively, this suggests that a significant fraction of SMARCAD1 functions in conjunction with architectural proteins in human cells.

### 3.2. Human TFIIIC102 Interacts Directly with SMARCAD1

**Figure 3:**
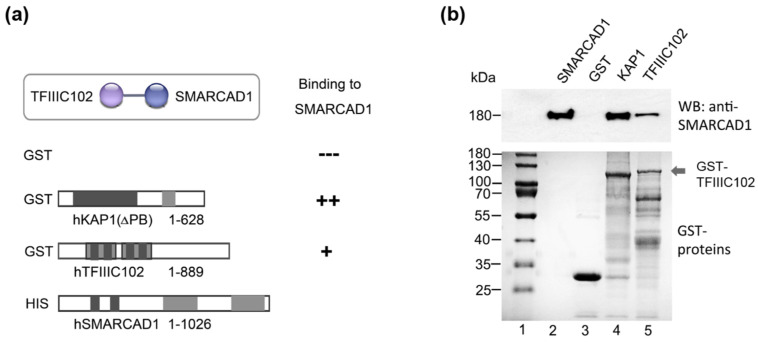
hTFIIIC102 binds directly to hSMARCAD1. (**a**) Schematic representation of the GST fusion proteins tested for interaction with His-SMARCAD1. Apart from KAP1 (Materials and Methods), all proteins were full-length. Amino acid numbers are given. (**b**) GST-pulldown assay. Top panel: Bound proteins detected by Western blot with an anti-SMARCAD1 antibody. His-SMARCAD1 expressed in insect cells (Input, lane 2, 20%), GST (lane 3), GST-KAP1 ΔPB (lane 4), and GST-TFIIIC102 (lane 5). Molecular weight markers are indicated (kDa). Bottom panel: Instant-blue-stained PAGE gels of purified GST proteins.

We wanted to confirm the interaction of the SMARCAD1 remodeler with TFIIIC in vitro and chose to focus on TFIIIC102. This subunit stands out because a link between TFIIIC102 and the SMARCAD1 homolog has been reported in *S. pombe* [39]. Furthermore, the TFIIIC102 subunit shows the highest evolutionary sequence conservation of the otherwise rather poorly conserved TFIIIC complex subunits [13,70,71]. To determine whether TFIIIC102 directly binds to SMARCAD1, we conducted GST-pulldown assays with recombinant, purified human proteins (Figure 3). SMARCAD1, expressed and purified via a His-tag from insect cells, was the bait (Figure 3b, Input, lane 2). We used full-length TFIIIC102 produced in bacteria as a GST fusion (Figure 3, cartoon and lane 5). As controls, GST-KAP1 (Figure 3b, lane 4), which is a strong physical interactor of SMARCAD1 [57,59], as well as GST alone (Figure 3b, lane 3) were utilized. Purified SMARCAD1 showed clear binding to the purified 102 kDa subunit of TFIIIC (Figure 3, lane 5). We conclude that the physical interaction between these two proteins is direct and not mediated by other factors. Since recombinant TFIIIC102 was expressed in bacteria, mammalian-specific posttranslational modifications (PTMs) of this protein are not absolutely required for this interaction. It remains possible that PTMs could affect the affinity of this interaction in vivo.

In sum, our data show that human SMARCAD1 and TFIIIC proteins interact in vitro and in vivo.

### 3.3. SMARCAD1 Interaction with TFIIIC Is Conserved in Pluripotent Mouse Cells

**Figure 4:**
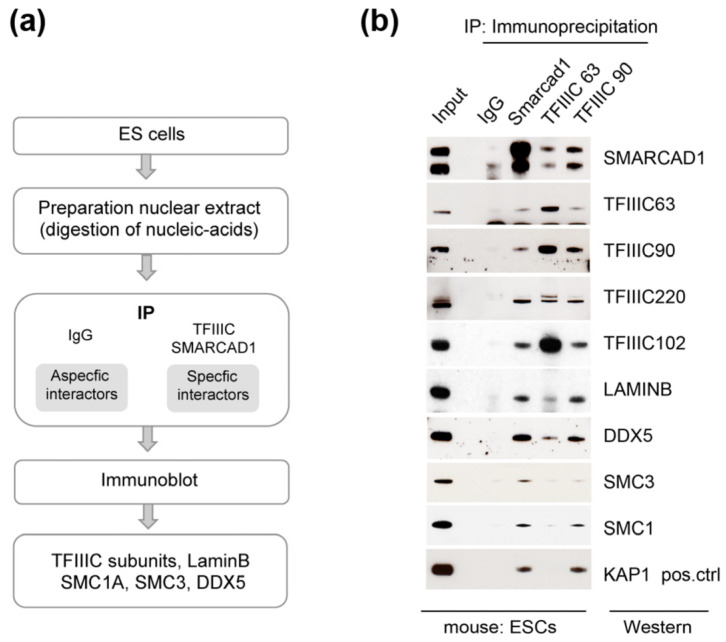
Architectural proteins associate with SMARCAD1 in mouse pluripotent ESCs. (**a**) Experimental set-up. (**b**) Western blot analysis of endogenous immunoprecipitates (IPs) from 46C nuclear extracts; 6% of nuclear extract was used as input. The IPs were performed in the presence of ethidium bromide and benzonase to exclude associations mediated by nucleic acids. SMARCAD1 runs as a doublet in these cells. The lower band in the TFIIIC63 panel corresponds to the heavy chain of the antibody used in the IP.

The protein interaction network of mammalian SMARCAD1 is likely to comprise interactions that are conserved and interactors that are distinct across different cell types and developmental stages to generate functional specificity. It raises the question of whether TFIIIC is a cell-type-specific or constitutive interaction partner of SMARCAD1. To address this, we chose mouse ESCs as a biological relevant cell system based on the following considerations: One, SMARCAD1 is highly expressed in stem cells and is downregulated upon differentiation [48,51,72]. Two, the genome-wide binding profile of SMARCAD1 has been determined in mESCs [48,51]. Three, mESCs display a unique chromatin landscape that distinguishes them from somatic cells such as those analyzed above [73,74]. Typical characteristics of mESCs include a relative open chromatin conformation with high transcriptional activity, whereas their differentiation is accompanied by an increase in heterochromatin and changes in higher-order genome organization [72,73,74,75]. Given these fundamental differences between pluripotent and differentiated cells, we wondered whether SMARCAD1 association with TFIIIC and chromatin architectural proteins observed in HEK293 and HeLa cells is conserved in mESCs. We therefore conducted endogenous co-immunoprecipitation experiments using antibodies against SMARCAD1, TFIIIC63, and TFIIIC90 in mESCs (Figure 4). Immunoblotting revealed that TFIIIC, monitored by antibodies to subunits 220, 102, 90, and 63, as well as SMC1A, SMC3, and DDX5, are bona fide, stable SMARCAD1-associated factors in pluripotent mESCs. So is laminB1, a major structural component of nuclear lamina that was identified as a candidate SMARCAD1 interactor in human SMARCAD1 IP-MS (Figure 1a) [57,61]. We further verified the association between SMARCAD1 and TFIIIC by reciprocally pulling down SMARCAD1 with antibodies against TFIIIC subunits 63 and 90. The results obtained were reminiscent of co-immunoprecipitation experiments performed in human somatic cells, which argues against a tissue specific role of the interaction between SMARCAD1, TFIIIC, and architectural proteins.

**Figure 5:**
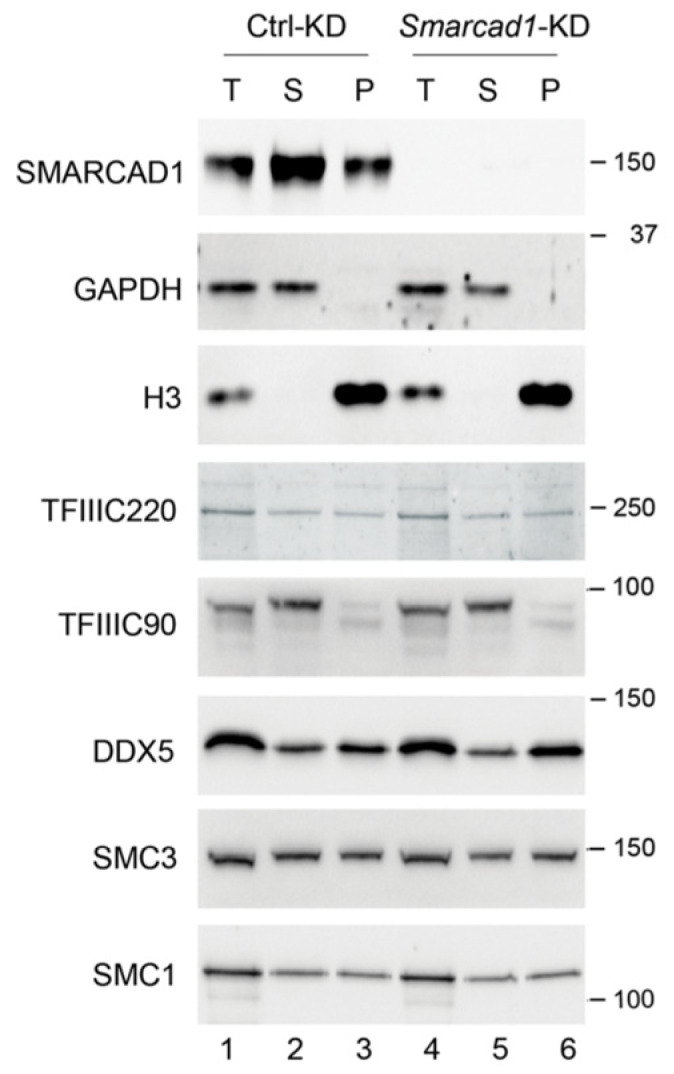
Total (T), soluble (S for supernatant), and chromatin (P for pellet) fractions were prepared from control (Ctrl) and stable *Smarcad1* knockdown (KD) ESCs (PGK12.1) and analyzed by Western blot with indicated antibodies. GAPDH and histone H3 were used as control for cytoplasm- and chromatin-bound fractions, respectively. Molecular weight markers are indicated in kDa. For TFIIIC90, bands of different mobility were detected, likely corresponding to differently modified versions of the protein.

Next, we performed cellular fractionation of ESCs (Figure 5). After extraction with detergent, proteins were separated into soluble (S for supernatant) and chromatin-enriched fractions (P for pellet), followed by Western blotting to visualize the distribution of architectural proteins. Except for TFIIIC90, which was readily extractable, leaving little protein in the pellet, about 50% of each protein analyzed remained in the chromatin enriched fraction in control cells. We then addressed whether knockdown of SMARCAD1 causes a change in either the amount or the localization of this set of SMARCAD1-interacting proteins, reflected in alterations in their relative abundance in the sub-cellular fractions. When we measured global cellular protein levels of TFIIIC90, TFIIIC220, cohesin subunits, and DDX5 in response to SMARCAD1 loss, we observed no overt increase or decrease (Figure 5, compare lanes 1 and 4). The knockdown of Smarcad1 was very efficient; as shown by immunoblot, the protein was not readily detectable (Figure 5, top panel, compare lane 1–3 with 4–6). However, this had no impact on the distribution of the analyzed proteins, suggesting that the SMARCAD1 remodeler does not play a major role in regulating the chromatin association of these proteins, at least at the global level, as judged by ease of extraction. It remains open whether SMARCAD1 influences chromatin occupancy of these factors at a subset of individual sites.

Collectively, our data reveal an association between SMARCAD1 and TFIIIC along with architectural proteins in multiple mammalian cell types, including pluripotent and somatic cells, pointing to a conserved role.

### 3.4. Impact of SMARCAD1 on the Expression of TFIIIC and TFIIIC Targets

**Figure 6:**
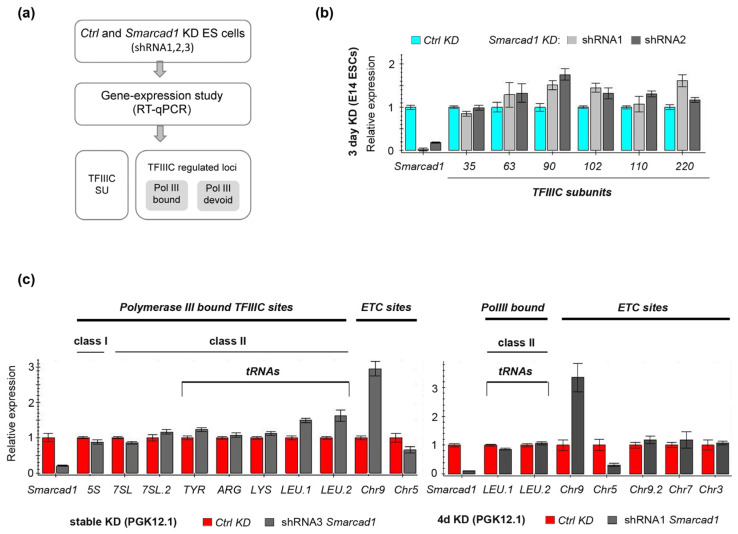
Gene expression analysis after depletion of *Smarcad1* in mESCs. (**a**) Experimental outline for testing the ability of SMARCAD1 to affect the transcription of TFIIIC- and TFIIIC-regulated loci. Two ESC lines were investigated, namely PGK12.1 and E14. SU, subunits. (**b**,**c**) RT-qPCR-based characterization of the relative expression of (**b**) TFIIIC subunits and (**c**) selected loci corresponding to different TFIIIC binding sites in control or *Smarcad1* knockdown ESCs. Gene expression is normalized to the average of two housekeeping genes and is presented as mean ± S.E. (error bar) of three technical triplicates. Biological replicates of datasets for b and c are shown in Supplementary Figure S2.

Having demonstrated the conservation of the physical interaction of SMARCAD1 with a central component of the RNA polymerase III transcription machinery, we went on to investigate whether this partnership is important for TFIIIC function in polymerase III transcription. In general, ATP-dependent chromatin remodelers are known to participate in the regulation of transcription, and SMARCAD1 has been characterized as both a transcriptional repressor, notably in ESCs, and a transcriptional activator of polymerase-II-controlled genes [51,76,77]. We investigated how SMARCAD1 might affect TFIIIC-dependent transcription in ESCs by examining the effect of *Smarcad1* knockdown (KD) on selective gene expression by RT-qPCR (Figure 6a).

First, we measured the mRNA levels of the six TFIIIC subunits themselves, as TFIIIC expression is altered in various diseases [78,79,80]. After performing RNAi with different *Smarcad1* shRNAs, we observed overall little change in the levels of TFIIIC transcripts in *Smarcad1* KD compared to control cells, apart from modest fluctuations (Figure 6a,b; Supplementary Figure S2a). This is consistent with the protein levels of TFIIIC220 and TFIIIC90 being unchanged following SMARCAD1 depletion (see Figure 5).

To determine if SMARCAD1 activity impacts the expression of TFIIIC-regulated loci, we carried out a comprehensive analysis of TFIIIC targets after exposing female and male mESCs to three different *Smarcad1*-specific shRNAs and examining the response of the cells to either stable or transient depletion of SMARCAD1. TFIIIC targets fall into several categories based on their particular combination of binding motifs and the presence of other polymerase III transcription factor complex components [14,15,16,81]. We chose representative sequences from different categories of TFIIIC-binding sites for closer interrogation. These include canonical targets of RNA Pol III, such as 5S and tRNA genes, as well as TFIIIC-bound regions in the genome that are not transcribed by RNA polymerase III, i.e., ETC sites [18]. The levels of 5S rRNA transcripts, which represent the RNA III polymerase Type I promoter, were not altered upon stable (Figure 6c, left panel) or transient (Supplementary Figure S2c) *Smarcad1* KD compared to control cells. Another RNA polymerase III transcript that requires TFIIIC, 7SL [82], also did not show any change in expression upon SMARCAD1 knockdown (Figure 6c, left panel). The RNA polymerase III Type II promoter, used most prominently by tRNA genes, harbors intragenic, highly conserved A and B boxes whose recognition by TFIIIC ultimately directs RNA polymerase recruitment to initiate transcription [15,16]. The relative abundance of individual tRNAs varies depending on the tissue or cell line and the physiological condition. In vertebrates, only a fraction of tRNA genes (about 60%) are bound by TFIIIC. Moreover, TFIIIC is absent from tRNAs not bound by polymerase III [22,83,84,85]. For our study, we selected tRNA genes documented in a genome-wide binding study in mESCs as being bound by TFIIIC and RNA polymerase III [21]. We found that their transcript levels were not significantly affected by SMARCAD1 knockdown (Figure 6c; Supplementary Figure S2b,c). Although some fluctuations were apparent, these were small and not robust across all conditions tested. For example, the tRNA LEU transcript levels were 1.3–1.5-fold higher in stable SMARCAD1 knockdown cells compared to control (Figure 6c, left panel). Yet, exposure of the same ESCs to the same or a different shRNA for four days did not result in increased levels (Figure 6c, right panel; Supplementary Figure S2b), and neither did SMARCAD1 depletion in another ESC line (Supplementary Figure S2c). Therefore, the observed increase in tRNA LEU may reflect differences in promoter accessibility or regulation caused by adaptation of this cell line to the permanent knockdown of *Smarcad1*. Overall, SMARCAD1 activity plays no major role in the control of the tRNA genes under study, as neither transient nor stable knockdown of *Smarcad1* resulted in consistent or dramatic changes in their expression. Moreover, cell-line-specific differences were not apparent based on comparing expression patterns between two different ESC lines. Collectively, these results suggest that SMARCAD1 depletion does not disrupt the proper expression of typical polymerase-III-bound genes.

Next, we turned to extra-TFIIIC sites. We selected five examples from a published list of ETC sites defined as TFIIIC-associated regions in mESCs not bound by the RNA polymerase III subunit RPC4 [21]. Of these five ETC sites, expression of three was unchanged, one was robustly up-regulated (ETC on chromosome 9 located in the intron of the *vwa9* gene), and one consistently downregulated (ETC on chromosome 5) upon SMARCAD1 depletion compared to the control, irrespective of the shRNA or the mESC line used for knockdown (Figure 6c; Supplementary Figure S2b,c). This suggests that SMARCAD1 participates in the regulation of a subset of ETC sites in the ESC genome. Inspection of the two affected ETC loci in the IGV genome browser showing SMARCAD1 ChIP-seq data from ESCs [51] revealed no called SMARCAD1 peaks over these sites, suggesting that the effect is indirect rather than direct. The underlying mechanistic details remain to be investigated in follow up studies, but it is unlikely to involve polymerase III.

Taken together, our loss-of-function studies suggest that SMARCAD1 does not function as a general regulator of the expression of TFIIIC-bound polymerase III sites in mESCs. We therefore favor a model whereby SMARCAD1 activity in mammals is involved in extra-transcriptional functions of TFIIIC, which is in line with our discovery of chromatin architectural proteins as conserved SMARCAD1 interactors.

### 3.5. SMARCAD1 and TFIIIC Binding Sites Are Distinct in the ESC Genome

**Figure 7:**
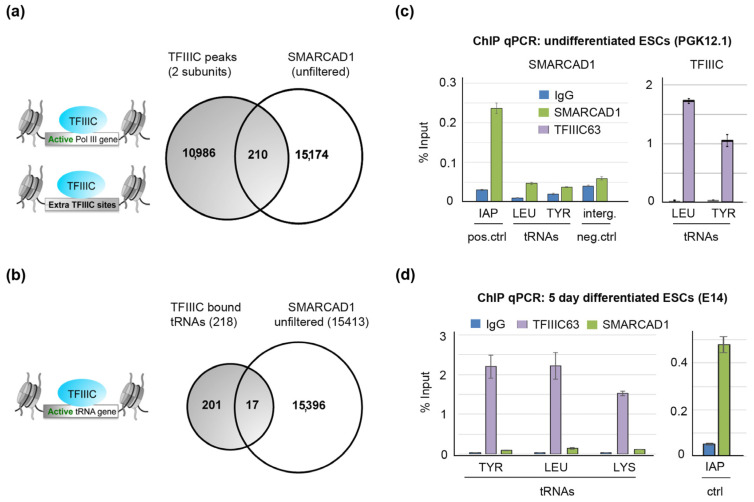
In mESCs, there is no enrichment of SMARCAD1 at TFIIIC sites. (**a**) Genome-wide, there is no significant overlap between an unfiltered ChIP-seq dataset of SMARCAD1 [51] and common peaks of TFIIIC110 and TFIIIC 220 [21] that mark active Pol III genes and extra-TFIIIC (ETC) sites. (**b**) Venn diagram showing the overlap of TFIIIC-bound tRNA genes with unfiltered SMARCAD1 ChIP-seq peaks in ESCs. (**c**,**d**) SMARCAD1 and TFIIIC enrichment (% of input) at tRNA genes in either undifferentiated (PGK12.1, Panel (**c**)) or 5-day-differentiated mESCs (E14, Panel (**d**)). The specificity of SMARCAD1 ChIP was evaluated using as a positive control intracisternal particle A (IAPez) and as a negative control an intergenic region. Percentage of input values are the mean ± S.E. of three technical replicates.

In *S. pombe*, the genomic sites occupied by SMARCAD1 (Fft3) overlap strongly with the TFIIIC-binding profile, in particular with actively transcribed polymerase III loci such as tRNA genes [39]. To investigate the possible chromatin co-occupancy of the mammalian counterparts of these proteins, we analyzed publicly available datasets obtained from ChIP-sequencing studies in the mESC genome. Around 260 tRNA genes and more than 2233 ETC loci were reported to be bound by TFIIIC in mESCs [21], and examples of tRNA genes on chromosome 11 bound by the polymerase III machinery as visualized in the genome browser IGV are shown in Supplementary Figure S3. SMARCAD1 was previously shown to bind predominantly to heterochromatin marked by H3K9me3 and KAP1, such as LTR retrotransposons [51]. TFIIIC and SMARCAD1 peaks were filtered such that only peaks were retained that had a minimum of 30 effective foreground reads and showed at least a 2.5-fold increase in the normalized read counts (TPM) compared to their backgrounds. When the genome-wide distribution of filtered SMARCAD1 [51] and TFIIIC [21] peaks were compared, the vast majority of binding sites for each protein were distinct with no significant overlap. Therefore, we changed two parameters: One, we expanded common peaks of TFIIIC110 and TFIIIC220 [21] by +/−500 bp to include possible SMARCAD1-binding sites in proximity of TFIIIC-bound sites. Two, rather than using filtered binding sites, we utilized unfiltered SMARCAD1 ChIP-seq data for comparison (Figure 7a). Even then, surprisingly little overlap between SMARCAD1 and expanded TFIIIC bound peaks was detected, namely 210 peaks from datasets with over 11,000 peaks.

We inspected 10% of those hits falling in the intersection in the genome browser IGV and analyzed whether there is a convincing overlap. Visual inspection of this randomly chosen subset of peaks did not reveal convincing overlapping peaks, as exemplified in Supplementary Figure S3.

We next focused specifically on tRNA genes to establish whether these fall into the intersection. We detected little overlap between SMARCAD1 and TFIIIC-bound tRNAs (Figure 7b, 17 peaks overlap); in fact, this overlap became zero when we used a high-confidence filtered SMARCAD1 binding site dataset defined as the common peaks of two separate ChIP-seq experiments detecting tagged, overexpressed, and endogenous SMARCAD1 proteins [51]. As the genome-wide binding datasets for TFIIIC and SMARCAD1 are derived from different mESC lines, we next performed ChIP with SMARCAD1 and TFIIIC antibodies in the same mESC line in parallel and carried out PCR amplification of tRNA genes (Figure 7c). While we could readily detect tDNA following precipitation with TFIIIC antibodies, we could not detect tRNA genes after precipitation with anti-SMARCAD antibodies. Importantly, in the SMARCAD1 ChIP sample, we amplified a known SMARCAD1 binding site successfully by qPCR (Figure 7c, positive control). Similar results were obtained in a second mESC line differentiated for five days (Figure 7d). Therefore, the lack of SMARCAD1 at tDNAs is not likely to be a consequence of suboptimal chromatin shearing, inefficient protein precipitation, poor primer sets, or differences in tRNA expression between mESC lines. Rather, these experiments support the notion that in pluripotent mouse cells, SMARCAD1 is not enriched at active tRNA genes, which is in stark contrast to the situation in budding and fission yeast [39,42]. Whether this is a characteristic unique to mESCs or also true of somatic mammalian cells remains to be determined, as the lower levels of SMARCAD1 expression in somatic cells has thwarted ChIP-seq attempts to date.

In conclusion, while SMARCAD1 and TFIIIC proteins interact in vitro and in nuclear extracts, unexpectedly, neither genome-wide analysis nor ChIP-qPCR experiments support their stable co-localization in ESC chromatin, opening up a number of possible speculative explanations discussed below.

## 4. Discussion

Members of the Fun30/Fft3/SMARCAD1 family of chromatin remodelers have important roles in genome stability and gene regulation. These functions of SMARCAD1 in mouse and human cells were previously explained in terms of the ability of SMARCAD1 to modulate chromatin organization locally. Here, we experimentally confirm an association of SMARCAD1 with complexes involved in chromatin insulation, three-dimensional folding of chromatin, and organization of the chromatin fiber within the nuclear space. This points to a novel role of the mammalian SMARCAD1 enzyme in chromosome organization in cooperation with architectural proteins.

The yeast homologs of SMARCAD1 have been implicated in chromatin insulation. Fft3 protects centromeres and sub-telomers [31,39], while the budding yeast homolog binds the silent mating-type locus HMR [40,42]. Deletion of Fft3 or Fun30 causes an altered chromatin structure at these barriers [31,39,40,42]. In mammals, SMARCAD1 is a critical component of the machinery that establishes and maintains heterochromatin domains characterized by tri-methylation of histone 3 at lysine 9 [51,52,57]. Its activity is required for the repression of endogenous and incoming exogenous retroviruses in mESCs, primarily modulating the local nucleosome structure [51,52,53,57,59]. Moreover, SMARCAD1 appears to remodel nucleosomes at sites of DNA damage to allow resection [86,87]. In line with these biological roles, an earlier proteomic analysis of SMARCAD1 uncovered a network of interactions with DNA-repair proteins and factors involved in transcriptional repression, including histone deacetylases and silencing factors such as KAP1 [57,59]. Here, we report the association of SMARCAD1 with TFIIIC, cohesin subunits SMC1A and SMC3, DDX5, and laminB in multiple mammalian cell lines and across different stages of cell differentiation. We found that KAP1, a stoichiometric component of SMARCAD1 complexes, likewise co-precipitates with TFIIIC, albeit specifically with components of the subcomplex B in HeLa and mESCs. What these architectural proteins have in common is their engagement in insulator function and higher-order chromatin organization [1,8,88]. For example, cohesin, best known for facilitating cohesion between sister chromatids, acts in loop-extrusion during interphase. It has been shown to be associated with TFIIIC in many species, and mutations in cohesin subunits compromise insulator function [19,21,22,27,33,64]. The distribution of these architectural proteins in different cellular fractions shows no overt change upon depletion of SMARCAD1. This argues against a direct role of SMARCAD1 in the chromatin recruitment or displacement of these factors in favor of an alternative model whereby SMARCAD1 is involved in modulating long-range interactions in mammalian genomes. We speculate that this may be related to the silencing function of this remodeler. An exciting possibility to be tested in the future is that SMARCAD1 impacts on TFIIIC and cohesin-dependent chromatin looping to join distant elements of the genome in order to regulate transcriptional silent domains.

In this study, we focused on the interaction of SMARCAD1 with TFIIIC, which we found is direct. An association between these factors has previously also been reported in fission yeast [39]. Such preservation of a partnership between a chromatin remodeler and a general transcription factor across diverse species and cell types with different chromatin features is indicative of a conserved role. TFIIIC has dual roles in chromatin organization and in RNA polymerase III transcription. Since ATP-dependent chromatin remodelers can impede or promote the binding of transcriptional regulators, it was anticipated that the Fun30/Fft3/SMARCAD1 family of remodelers impact, transcription. They have been reported to contribute to the initiation and the elongation stages of transcription mediated by polymerase II [76,77,89]. The current study suggests that SMARCAD1 does not regulate RNA polymerase III transcription per se in mESCs. In our analysis of typical polymerase III targets, SMARCAD1 was not critical for the transcription function of TFIIIC. Moreover, we did not find evidence that SMARCAD1 binds to actively transcribed TFIIIC-bound polymerase III sites, such as tRNA genes. This represents a significant difference from the situation in yeast where the SMARCAD1 homologs of both fission and budding yeast are clearly enriched at active tRNA genes [39,42]. In the case of Fft3, it was suggested that it affects the transcription of some but not all tRNA classes based on the analysis of two tRNA genes, but it is not understood how [39]. The second major function of TFIIIC observed throughout eukaryotic evolution is in chromatin insulation [13,18,20,26,27,38,90]. Nucleosome depletion at tDNAs is a recurrent feature in this and is believed to assist stable TFIIIC binding and to block spreading of chromatin states. The action of chromatin remodeling complexes is likely relevant in this context, as has been demonstrated for the SWI/SNF remodeler RSC (Remodels the Structure of Chromatin) in budding yeast [90,91]. Subunits of human SWI/SNF complexes have since been found selectively enriched at tDNA, but functional evidence has yet to be obtained for their involvement in chromatin insulation [13,92]. In vitro, the SMARCAD1 enzyme can assemble and disassemble nucleosomes [93], but whether it functions in vivo at tRNA genes in some mammalian cell types needs clarification. In many species, TFIIIC controls the intranuclear localization of its targets, including tRNA genes, which are frequently associated with nuclear pores [18,20,28,37]. Interestingly, in cells lacking Fft3, insulators and sub-telomeres move away from the nuclear envelope, suggesting that remodeling by Fft3 has a role in anchoring these regions [39]. Therefore, the possibility of a cooperation of SMARCAD1 with TFIIIC in tethering chromatin domains to nuclear substructures to provide a transcriptional unfavorable environment warrants future investigation.

The localization of SMARCAD1 in the mESC genome depends largely on its interaction partner KAP1 [59]. Because SMARCAD1 does not harbor a sequence-specific DNA binding domain, it is likely that other SMARCAD1 interactors help recruit it to other sites. We have shown here that SMARCAD1 makes direct contact with at least one TFIIIC subunit, TFIIIC102. The same subunit was discovered as a partner of Fft3 in a yeast-two-hybrid screen [39]. With its intrinsic DNA-sequence-specific binding capability, the TFIIIC complex represents a good candidate for a SMARCAD1/Fft3 chromatin recruitment factor. In agreement with this, there is strong overlap between the Fft3, TFIIIC, and RNA polymerase III binding profiles in fission yeast [31]. Yet, in the chromatin of pluripotent mESCs, we detected no robust co-localization of SMARCAD1 and TFIIIC, suggesting that their interaction demonstrated in mESC extracts does either not occur on chromatin of normal cycling mESCs or is transient. We focused our analysis on pluripotent cell chromatin, as SMARCAD1 and TFIIIC have both been suggested to play a role in stem cell homeostasis [48,50,72,94,95]. However, mESCs have an unusual and dynamic chromatin configuration [72,73,74], which may represent a special case for the SMARCAD1–TFIIIC relationship. Lineage commitment upon mESC differentiation is accompanied by the gain of more loop-structures, changes in laminB–chromatin interactions, and an increase in heterochromatin [9,63,74,75,96].

It remains to be seen whether SMARCAD1 and TFIIIC co-occupy genomic sites in somatic cells, where we also demonstrated a robust association between these factors. Moreover, it remains open whether they co-localize on chromatin only under distinct physiological conditions. One possibility is that the TFIIIC/SMARCAD1 complex that we have characterized could be sequestered in the nucleus at non-chromatin sites and in response to an appropriate signal associate with specific genomic loci. Interestingly, TFIIIC has cell-type-specific functions and can be redistributed in the genome depending on the particular conditions, e.g., stress, to mediate gene regulation via chromatin looping [35,36]. For instance, in T47D breast cancer cells, serum starvation triggers TFIIIC accumulation at Alu repeats, accompanied by an increase in histone acetylation, which favors transcription [35]. In murine neurons, TFIIIC binding to short interspersed nuclear elements (SINEs) regulates activity-dependent transcription through rearrangement of nuclear architecture [97]. These and other studies illustrate that in human and mouse cells, TFIIIC can associate with repetitive sequences, several of which participate in 3D genome organization [35,36,84,97,98,99]. This may be relevant given that we have previously shown that SMARCAD1 binds predominantly to repetitive elements in the mESCs genome [51].

Future studies using imaging and Hi-C-related methods are needed to delineate the significance of the mammalian TFIIIC/SMARCAD1 partnership and determine if it shapes genome architecture together with specific repetitive elements.

## Data Availability

SMARCAD1 sequencing datasets were generated in our laboratory [51]. TFIIIC ChIP-seq datasets were downloaded from [21].

## Supplementary Materials

The following supporting information can be downloaded at: https://www.mdpi.com/article/10.3390/genes14091793/s1. Figure S1: Peptides corresponding to architectural proteins associated with human SMARCAD1. Figure S2: Relative expression of selected genes corresponding to the six TFIIIC subunits or different TFIIIC-binding sites after control or *Smarcad1* shRNA-mediated knockdown. Figure S3: A tRNA cluster at chromosome 11 is bound by TFIIIC and RNA polymerase III but devoid of SMARCAD1 enrichment. Table S1: Primer table.
