# Supplementary material for: The Conserved Chromatin Remodeler SMARCAD1 Interacts with TFIIIC and Architectural Proteins in Human and Mouse
Source: Genes (Basel). 2023 Sep 13;14(9):1793. doi: 10.3390/genes14091793 (PMC10530723; doi:10.3390/genes14091793)
Supplement: Supplementary file 1 [file genes-14-01793-s001.zip › genes-2599697-figure S1.pdf]

Figure S1: related to Figure 1
Figure S2: related to Figure 6
Figure S3: related to Figure 7
Table S1: Primers used in Expression and ChIP-qPCR Experiments.

Figure S1: related to Figure 1
Peptides corresponding to architectural proteins associated with human SMARCAD1. Shown are the sequences of proteins validated in co-immunoprecipitation experiments; underlined are the peptides identified in a FLAG-SMARCAD1 pull down from HEK293 cells by label free mass spectrometry [1].
>sp|Q12789|TF3C1_HUMAN General transcription factor 3C polypeptide 1
OS=Homo sapiens OX=9606 GN=GTF3C1 PE=1 SV=4
MDALESLLDEVALEGLDGLCLPALWSRLETRVPPFPLPLEPCTQEFLWRALATHPGISFY
EEPRERPDLQLQDRYEEIDLETGILESRRDPVALEDVYPIHMILENKDGIQGSCRYFKER
KNITNDIRTKSLQPRCTMVEAFDRWGKKLIIVASQAMRYRALIGQEGDPDLKLPDFSYCI
LERLGRSRWQGELQRDLHTTAFKVDAGKLHYHRKILNKNGLITMQSHVIRLPTGAQQHSI
LLLLNRFHVDRRSKYDILMEKLSVMLSTRTNHIETLGKLREELGLCERTFKRLYQYMLNA
GLAKVVSLRLQEIHPECGPCKTKKGTDVMVRCLKLLKEFKRNDHDDDEDEEVISKTVPPV
DIVFERDMLTQTYDLIERRGTKGISQAEIRVAMNVGKLEARMLCRLLQRFKVVKGFMEDE
GRQRTTKYISCVFAEESDLSRQYQREKARSELLTTVSLASMQEESLLPEGEDTFLSESDS
EEERSSSKRRGRGSQKDTRASANLRPKTQPHHSTPTKGGWKVVNLHPLKKQPPSFPGAAE
ERACQSLASRDSLLDTSSVSEPNVSFVSHCADSNSGDIAVIEEVRMENPKESSSSLKTGR
HSSGQDKPHETYRLLKRRNLIIEAVTNLRLIESLFTIQKMIMDQEKQEGVSTKCCKKSIV
RLVRNLSEEGLLRLYRTTVIQDGIKKKVDLVVHPSMDQNDPLVRSAIEQVRFRISNSSTA
NRVKTSQPPVPQGEAEEDSQGKEGPSGSGDSQLSASSRSESGRMKKSDNKMGITPLRNYH
PIVVPGLGRSLGFLPKMPRLRVVHMFLWYLIYGHPASNTVEKPSFISERRTIKQESGRAG
VRPSSSGSAWEACSEAPSKGSQDGVTWEAEVELATETVYVDDASWMRYIPPIPVHRDFGF
GWALVSDILLCLPLSIFIQIVQVSYKVDNLEEFLNDPLKKHTLIRFLPRPIRQQLLYKRR
YIFSVVENLQRLCYMGLLQFGPTEKFQDKDQVFIFLKKNAVIVDTTICDPHYNLARSSRP
FERRLYVLNSMQDVENYWFDLQCVCLNTPLGVVRCPRVRKNSSTDQGSDEEGSLQKEQES
AMDKHNLERKCAMLEYTTGSREVVDEGLIPGDGLGAAGLDSSFYGHLKRNWIWTSYIINQ
AKKENTAAENGLTVRLQTFLSKRPMPLSARGNSRLNIWGEARVGSELCAGWEEQFEVDRE
PSLDRNRRVRGGKSQKRKRLKKDPGKKIKRKKKGEFPGEKSKRLRYHDEADQSALQRMTR
LRVTWSMQEDGLLVLCRIASNVLNTKVKGPFVTWQVVRDILHATFEESLDKTSHSVGRRA
RYIVKNPQAYLNYKVCLAEVYQDKALVGDFMNRRGDYDDPKVCANEFKEFVEKLKEKFSS
ALRNSNLEIPDTLQELFARYRVLAIGDEKDQTRKEDELNSVDDIHFLVLQNLIQSTLALS
DSQMKSYQSFQTFRLYREYKDHVLVKAFMECQKRSLVNRRRVNHTLGPKKNRALPFVPMS
YQLSQTYYRIFTWRFPSTICTESFQFLDRMRAAGKLDQPDRFSFKDQDNNEPTNDMVAFS
LDGPGGNCVAVLTLFSLGLISVDVRIPEQIIVVDSSMVENEVIKSLGKDGSLEDDEDEED
DLDEGVGGKRRSMEVKPAQASHTNYLLMRGYYSPGIVSTRNLNPNDSIVVNSCQMKFQLR
CTPVPARLRPAAAPLEELTMGTSCLPDTFTKLINPQENTCSLEEFVLQLELSGYSPEDLT
AALEILEAIIATGCFGIDKEELRRRFSALEKAGGGRTRTFADCIQALLEQHQVLEVGGNT
ARLVAMGSAWPWLLHSVRLKDREDADIQREDPQARPLEGSSSEDSPPEGQAPPSHSPRGT
KRRASWASENGETDAEGTQMTPAKRPALQDSNLAPSLGPGAEDGAEAQAPSPPPALEDTA
AAGAAQEDQEGVGEFSSPGQEQLSGQAQPPEGSEDPRGFTESFGAANISQAARERDCESV
CFIGRPWRVVDGHLNLPVCKGMMEAMLYHIMTRPGIPESSLLRHYQGVLQPVAVLELLQG
LESLGCIRKRWLRKPRPVSLFSTPVVEEVEVPSSLDESPMAFYEPTLDCTLRLGRVFPHE
VNWNKWIHL
>sp|Q8WUA4|TF3C2_HUMAN General transcription factor 3C polypeptide 2
OS=Homo sapiens OX=9606 GN=GTF3C2 PE=1 SV=2
MDTCGVGYVALGEAGPVGNMTVVDSPGQEVLNQLDVKTSSEMTSAEASVEMSLPTPLPGF
EDSPDQRRLPPEQESLSRLEQPDLSSEMSKVSKPRASKPGRKRGGRTRKGPKRPQQPNPP
SAPLVPGLLDQSNPLSTPMPKKRGRKSKAELLLLKLSKDLDRPESQSPKRPPEDFETPSG
ERPRRRAAQVALLYLQELAEELSTALPAPVSCPEGPKVSSPTKPKKIRQPAACPGGEEVD
GAPRDEDFFLQVEAEDVEESEGPSESSSEPEPVVPRSTPRGSTSGKQKPHCRGMAPNGLP

NHIMAPVWKCLHLTKDFREQKHSYWEFAEWIPLAWKWHLLSELEAAPYLPQEEKSPLFSV
QREGLPEDGTLYRINRFSSITAHPERWDVSFFTGGPLWALDWCPVPEGAGASQYVALFSS
PDMNETHPLSQLHSGPGLLQLWGLGTLQQESCPGNRAHFVYGIACDNGCIWDLKFCPSGA
WELPGTPRKAPLLPRLGLLALACSDGKVLLFSLPHPEALLAQQPPDAVKPAIYKVQCVAT
LQVGSMQATDPSECGQCLSLAWMPTRPHQHLAAGYYNGMVVFWNLPTNSPLQRIRLSDGS
LKLYPFQCFLAHDQAVRTLQWCKANSHFLVSAGSDRKIKFWDLRRPYEPINSIKRFLSTE
LAWLLPYNGVTVAQDNCYASYGLCGIHYIDAGYLGFKAYFTAPRKGTVWSLSGSDWLGTI
AAGDISGELIAAILPDMALNPINVKRPVERRFPIYKADLIPYQDSPEGPDHSSASSGVPN
PPKARTYTETVNHHYLLFQDTDLGSFHDLLRREPMLRMQEGEGHSQLCLDRLQLEAIHKV
RFSPNLDSYGWLVSGGQSGLVRIHFVRGLASPLGHRMQLESRAHFNAMFQPSSPTRRPGF
SPTSHRLLPTP
>sp|Q9Y5Q9|TF3C3_HUMAN General transcription factor 3C polypeptide 3
OS=Homo sapiens OX=9606 GN=GTF3C3 PE=1 SV=1
MSGFSPELIDYLEGKISFEEFERRREERKTREKKSLQEKGKLSAEENPDDSEVPSSSGIN
STKSQDKDVNEGETSDGVRKSVHKVFASMLGENEDDEEEEEEEEEEEEEEETPEQPTAGD
VFVLEMVLNRETKKMMKEKRPRSKLPRALRGLMGEANIRFARGEREEAILMCMEIIRQAP
LAYEPFSTLAMIYEDQGDMEKSLQFELIAAHLNPSDTEEWVRLAEMSLEQDNIKQAIFCY
TKALKYEPTNVRYLWERSSLYEQMGDHKMAMDGYRRILNLLSPSDGERFMQLARDMAKSY
YEANDVTSAINIIDEAFSKHQGLVSMEDVNIAAELYISNKQYDKALEIITDFSGIVLEKK
TSEEGTSEENKAPENVTCTIPDGVPIDITVKLMVCLVHLNILEPLNPLLTTLVEQNPEDM
GDLYLDVAEAFLDVGEYNSALPLLSALVCSERYNLAVVWLRHAECLKALGYMERAAESYG
KVVDLAPLHLDARISLSTLQQQLGQPEKALEALEPMYDPDTLAQDANAAQQELKLLLHRS
TLLFSQGKMYGYVDTLLTMLAMLLKVAMNRAQVCLISSSKSGERHLYLIKVSRDKISDSN
DQESANCDAKAIFAVLTSVLTKDDWWNLLLKAIYSLCDLSRFQEAELLVDSSLEYYSFYD
DRQKRKELEYFGLSAAILDKNFRKAYNYIRIMVMENVNKPQLWNIFNQVTMHSQDVRHHR
FCLRLMLKNPENHALCVLNGHNAFVSGSFKHALGQYVQAFRTHPDEPLYSFCIGLTFIHM
ASQKYVLRRHALIVQGFSFLNRYLSLRGPCQESFYNLGRGLHQLGLIHLAIHYYQKALEL
PPLVVEGIELDQLDLRRDIAYNLSLIYQSSGNTGMAQTLLYTYCSI
>sp|Q9UKN8|TF3C4_HUMAN General transcription factor 3C polypeptide 4
OS=Homo sapiens OX=9606 GN=GTF3C4 PE=1 SV=2
MNTADQARVGPADDGPAPSGEEEGEGGGEAGGKEPAADAAPGPSAAFRLMVTRREPAVKL
QYAVSGLEPLAWSEDHRVSVSTARSIAVLELICDVHNPGQDLVIHRTSVPAPLNSCLLKV
GSKTEVAECKEKFAASKDPTVSQTFMLDRVFNPEGKALPPMRGFKYTSWSPMGCDANGRC
LLAALTMDNRLTIQANLNRLQWVQLVDLTEIYGERLYETSYRLSKNEAPEGNLGDFAEFQ
RRHSMQTPVRMEWSGICTTQQVKHNNECRDVGSVLLAVLFENGNIAVWQFQLPFVGKESI
SSCNTIESGITSPSVLFWWEYEHNNRKMSGLIVGSAFGPIKILPVNLKAVKGYFTLRQPV
ILWKEMDQLPVHSIKCVPLYHPYQKCSCSLVVAARGSYVFWCLLLISKAGLNVHNSHVTG
LHSLPIVSMTADKQNGTVYTCSSDGKVRQLIPIFTDVALKFEHQLIKLSDVFGSVRTHGI
AVSPCGAYLAIITTEGMINGLHPVNKNYQVQFVTLKTFEEAAAQLLESSVQNLFKQVDLI
DLVRWKILKDKHIPQFLQEALEKKIESSGVTYFWRFKLFLLRILYQSMQKTPSEALWKPT
HEDSKILLVDSPGMGNADDEQQEEGTSSKQVVKQGLQERSKEGDVEEPTDDSLPTTGDAG
GREPMEEKLLEIQGKIEAVEMHLTREHMKRVLGEVYLHTWITENTSIPTRGLCNFLMSDE
EYDDRTARVLIGHISKKMNKQTFPEHCSLCKEILPFTDRKQAVCSNGHIWLRCFLTYQSC
QSLIYRRCLLHDSIARHPAPEDPDWIKRLLQSPCPFCDSPVF
>sp|Q9Y5Q8|TF3C5_HUMAN General transcription factor 3C polypeptide 5
OS=Homo sapiens OX=9606 GN=GTF3C5 PE=1 SV=2
MAAEAADLGLGAAVPVELRRERRMVCVEYPGVVRDVAKMLPTLGGEEGVSRIYADPTKRL
ELYFRPKDPYCHPVCANRFSTSSLLLRIRKRTRRQKGVLGTEAHSEVTFDMEILGIISTI
YKFQGMSDFQYLAVHTEAGGKHTSMYDKVLMLRPEKEAFFHQELPLYIPPPIFSRLDAPV
DYFYRPETQHREGYNNPPISGENLIGLSRARRPHNAIFVNFEDEEVPKQPLEAAAQTWRR
VCTNPVDRKVEEELRKLFDIRPIWSRNAVKANISVHPDKLKVLLPFIAYYMITGPWRSLW
IRFGYDPRKNPDAKIYQVLDFRIRCGMKHGYAPSDLPVKAKRSTYNYSLPITVKKTSSQL
VTMHDLKQGLGPSGTSGARKPASSKYKLKDSVYIFREGALPPYRQMFYQLCDLNVEELQK

IIHRNDGAENSCTERDGWCLPKTSDELRDTMSLMIRQTIRSKRPALFSSSAKADGGKEQL
TYESGEDEEDEEEEEEEEEDFKPSDGSENEMETEILDYV
>tr|Q68EN4|Q68EN4_HUMAN SMC1A protein (Fragment) OS=Homo sapiens
OX=9606 GN=SMC1A PE=2 SV=1
MGFLKLIEIENFKSYKGRQIIGPFQRFTAIIGPNGSGKSNLMDAISFVLGEKTSNLRVKT
LRDLIHGAPVGKPAANRAFVSMVYSEEGAEDRTFARVIVGGSSEYKINNKVVQLHEYSEE
LEKLGILIKARNFLVFQGAVESIAMKNPKERTALFEEISRSGELAQEYDKRKKEMVKAEE
DTQFNYHRKKNIAAERKEAKQEKEEADRYQRLKDEVVRAQVQLQLFKLYHNEVEIEKLNK
ELASKNKEIEKDKKRMDKVEDELKEKKKELGKMMREQQQIEKEIKEKDSELNQKRPQYIK
AKENTSHKIKKLEAAKKSLQNAQKHYKKRKGDMDELEKEMLSVEKARQEFEERMEEESQS
QGRDLTLEENQVKKYHRLKEEASKRAATLAQELEKFNRDQKADQDRLDLEERKKKKK
>sp|Q9UQE7|SMC3_HUMAN Structural maintenance of chromosomes protein 3
OS=Homo sapiens OX=9606 GN=SMC3 PE=1 SV=2
MYIKQVIIQGFRSYRDQTIVDPFSSKHNVIVGRNGSGKSNFFYAIQFVLSDEFSHLRPEQ
RLALLHEGTGPRVISAFVEIIFDNSDNRLPIDKEEVSLRRVIGAKKDQYFLDKKMVTKND
VMNLLESAGFSRSNPYYIVKQGKINQMATAPDSQRLKLLREVAGTRVYDERKEESISLMK
ETEGKREKINELLKYIEERLHTLEEEKEELAQYQKWDKMRRALEYTIYNQELNETRAKLD
ELSAKRETSGEKSRQLRDAQQDARDKMEDIERQVRELKTKISAMKEEKEQLSAERQEQIK
QRTKLELKAKDLQDELAGNSEQRKRLLKERQKLLEKIEEKQKELAETEPKFNSVKEKEER
GIARLAQATQERTDLYAKQGRGSQFTSKEERDKWIKKELKSLDQAINDKKRQIAAIHKDL
EDTEANKEKNLEQYNKLDQDLNEVKARVEELDRKYYEVKNKKDELQSERNYLWREENAEQ
QALAAKREDLEKKQQLLRAATGKAILNGIDSINKVLDHFRRKGINQHVQNGYHGIVMNNF
ECEPAFYTCVEVTAGNRLFYHIVDSDEVSTKILMEFNKMNLPGEVTFLPLNKLDVRDTAY
PETNDAIPMISKLRYNPRFDKAFKHVFGKTLICRSMEVSTQLARAFTMDCITLEGDQVSH
RGALTGGYYDTRKSRLELQKDVRKAEEELGELEAKLNENLRRNIERINNEIDQLMNQMQQ
IETQQRKFKASRDSILSEMKMLKEKRQQSEKTFMPKQRSLQSLEASLHAMESTRESLKAE
LGTDLLSQLSLEDQKRVDALNDEIRQLQQENRQLLNERIKLEGIITRVETYLNENLRKRL
DQVEQELNELRETEGGTVLTATTSELEAINKRVKDTMARSEDLDNSIDKTEAGIKELQKS
MERWKNMEKEHMDAINHDTKELEKMTNRQGMLLKKKEECMKKIRELGSLPQEAFEKYQTL
SLKQLFRKLEQCNTELKKYSHVNKKALDQFVNFSEQKEKLIKRQEELDRGYKSIMELMNV
LELRKYEAIQLTFKQVSKNFSEVFQKLVPGGKATLVMKKGDVEGSQSQDEGEGSGESERG
SGSQSSVPSVDQFTGVGIRVSFTGKQGEMREMQQLSGGQKSLVALALIFAIQKCDPAPFY
LFDEIDQALDAQHRKAVSDMIMELAVHAQFITTTFRPELLESADKFYGVKFRNKVSHIDV
ITAEMAKDFVEDDTTHG
>sp|P17844|DDX5_HUMAN Probable ATP-dependent RNA helicase DDX5
OS=Homo sapiens OX=9606 GN=DDX5 PE=1 SV=1
MSGYSSDRDRGRDRGFGAPRFGGSRAGPLSGKKFGNPGEKLVKKKWNLDELPKFEKNFYQ
EHPDLARRTAQEVETYRRSKEITVRGHNCPKPVLNFYEANFPANVMDVIARQNFTEPTAI
QAQGWPVALSGLDMVGVAQTGSGKTLSYLLPAIVHINHQPFLERGDGPICLVLAPTRELA
QQVQQVAAEYCRACRLKSTCIYGGAPKGPQIRDLERGVEICIATPGRLIDFLECGKTNLR
RTTYLVLDEADRMLDMGFEPQIRKIVDQIRPDRQTLMWSATWPKEVRQLAEDFLKDYIHI
NIGALELSANHNILQIVDVCHDVEKDEKLIRLMEEIMSEKENKTIVFVETKRRCDELTRK
MRRDGWPAMGIHGDKSQQERDWVLNEFKHGKAPILIATDVASRGLDVEDVKFVINYDYPN
SSEDYIHRIGRTARSTKTGTAYTFFTPNNIKQVSDLISVLREANQAINPKLLQLVEDRGS
GRSRGRGGMKDDRRDRYSAGKRGGFNTFRDRENYDRGYSSLLKRDFGAKTQNGVYSAANY
TNGSFGSNFVSAGIQTSFRTGNPTGTYQNGYDSTQQYGSNVPNMHNGMNQQAYAYPATAA
APMIGYPMPTGYSQ
