# Supplementary material for: The Conserved Chromatin Remodeler SMARCAD1 Interacts with TFIIIC and Architectural Proteins in Human and Mouse
Source: Genes (Basel). 2023 Sep 13;14(9):1793. doi: 10.3390/genes14091793 (PMC10530723; doi:10.3390/genes14091793)
Supplement: Supplementary file 1 [file genes-14-01793-s001.zip › genes-2599697-supplementary.pdf]

Supplementary Materials

Figure S1: related to Figure 1

Figure S2: related to Figure 6

Figure S3: related to Figure 7

Table S1: Primers used in Expression and ChIP-qPCR Experiments.

Figure S2: related to Figure 6

Relative expression of selected genes corresponding to the six TFIIIC subunits **(a)** or different TFIIIC binding sites **(b,c)** after control or *Smarcad1* shRNA-mediated knockdown. Shown is the fold-change of three replicates normalized to two-three housekeeping genes, error bar depicts ± SEM.

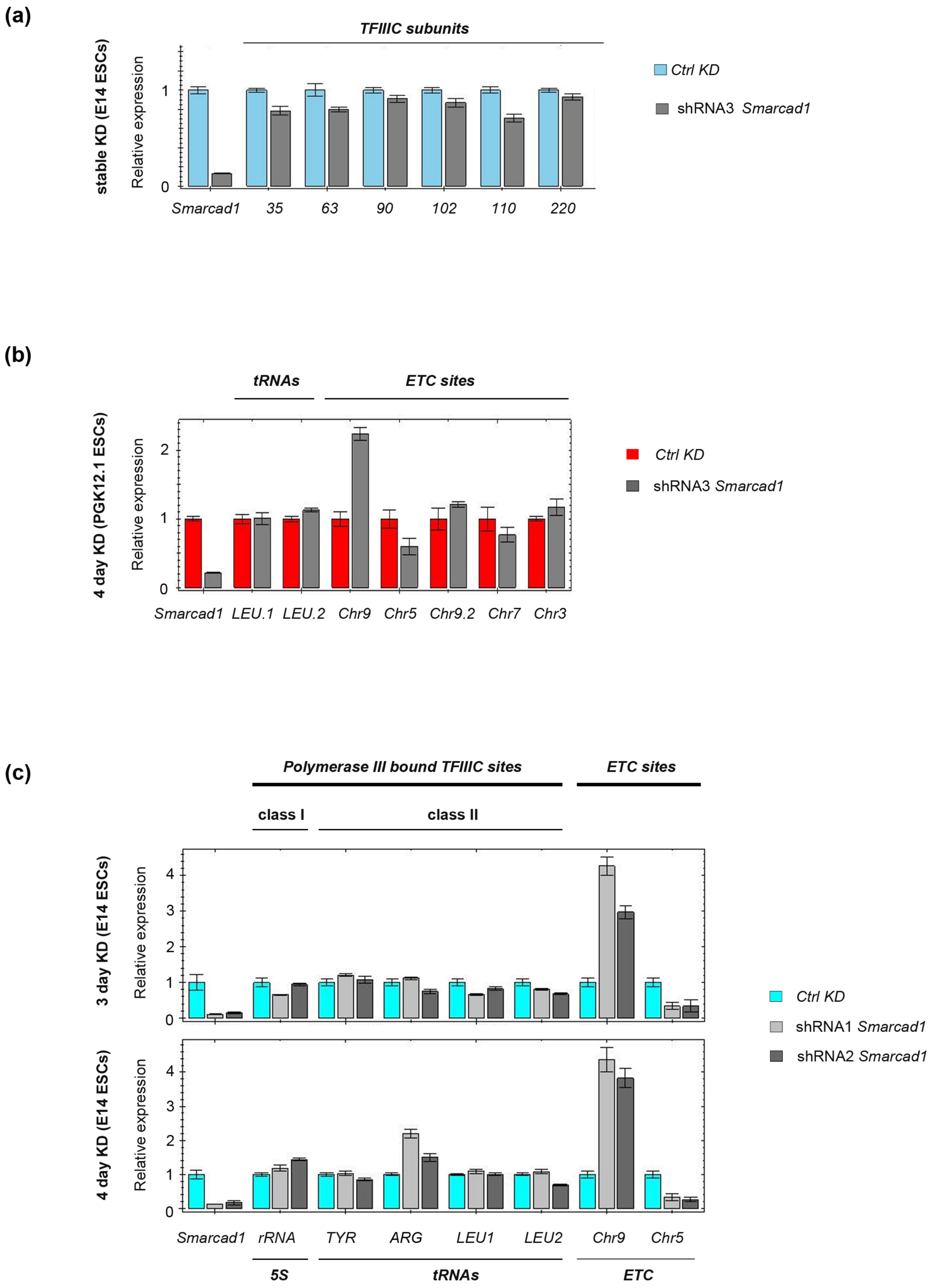

Figure S3: related to Figure 7

A tRNA cluster at chromosome 11 is bound by TFIIIC and RNA polymerase III but devoid of SMARCAD1 enrichment. Screenshot of a genome browser (IGV) view of published ChIP-seq peaks of the polymerase III apparatus [2] and SMARCAD1 [3] in ESCs (mm10). Indicated are individual tRNA genes (blue vertical lines) and selected peaks corresponding to the intersection between TFIIIC and SMARCAD1 peaks (blue boxes).

.

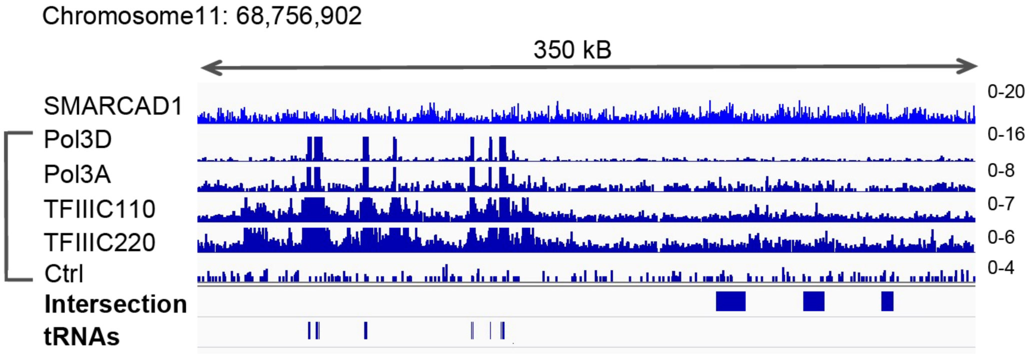

Table S1: Primers used in Expression and ChIP Experiments.

Target	Sequence (5′-3′) f and r	Application	Reference
TFIIIC35	GCGTCTCCGAACGCTATGG<br>CCTCTCCGTGTCAATTCCCA	RT-qPCR	[4]
TFIIIC63	TTGGTGTGCGTGGAGTACC<br>CCCAGGGTCTGTAGCATCT	RT-qPCR	[4]
TFIIIC90	CCCAATGCGGGGTTTCAAATA<br>CTGCACAGTCAGGCGATTGT	RT-qPCR	[4]
TFIIIC90	AAACAGAAGTTGCTGAGTGC<br>ATGGTCAGGCGATTGTCC	RT-qPCR	[5]
TFIIIC102	TGTGCAGGGCTTTTCCTTCC<br>TGAGCCATTGCAGTGTTCCC	RT-qPCR	this study
TFIIIC110	CCAGAAGGGGTCTCAAAAGTCC<br>CTTTCTTCAGAGATGTCAAAGG	RT-qPCR	[5]
TFIIIC220	TCCAGCGAGACCTTCACACC<br>GGATTGAGTGTTGCTGGGCT	RT-qPCR	[5]
Atp5b	GGCCAAGATGTCCTGCTGTT<br>GCTGGTAGCCTACAGCAGAAGG	RT-qPCR	[6]
GAPDH	TCCATGACAACTTTGGCATTG<br>CAGTCTTCTGGGTGGCAGTGA	RT-qPCR	[7]
SMARCAD1	TTCCTGGCATACCTCTTTC<br>ATTTGCTTACGCTCTTCTTG	RT-qPCR	[8]
5S rRNA	GGCCATACCACCCTGAACGC<br>CAGCACCCGGTATTCCCAGG	RT-qPCR	[9]
5S rRNA	TCTCGTCTGATCTCGGAAGC<br>AGCCTACAGCACCCGGTATT	RT-qPCR	[4]
7SL	ATCGGGTGTCCGCACTAAGTT<br>CAGCACGGGAGTTTTGACCT	RT-qPCR	[10]
7SL.2	GTGTCCGCACTAAGTTCGG<br>TATTCACAGGCGCGATCC	RT-qPCR	[4]
tRNA Leu.1 chr 11<br>chr11:48747685+48747778	ACAGCAACGGTCTTCACATAGC<br>GCCGTCCATTCTGAGATTCTGT	RT-qPCR	this study
tRNA Leu.2 chr 11<br>chr11:48747733+48747810	GCCACCTACCAGTCAATCGGA<br>ACCGCTGCCAGTAACTTTTGAG	RT-qPCR	this study
tRNA TYR	CCTTCGATAGCTCAGCTGGTAGAGCGGAGG<br>CGGAATTGAACCAGCGACCTAAGGATGTCC	RT-qPCR	[5]
tRNA ARG	CCGCGTGGCCTAATGGATAA<br>GACCACGAAGGGACTCGAAC	RT-qPCR	this study
tRNA LYS	GCCTGGATAGCTCAGTCGGT<br>CGCCTGAACAGGGACTTGA	RT-qPCR	this study
ETC chr9<br>chr9:64970923+64971131	GCCCAGAATCTGTACGTGGT<br>TCAACTCAGTCTGTGACCGC	RT-qPCR	this study
ETC chr5<br>chr5:147716219+147716388	CTACTTCAGGGCTCTTCGCAT<br>GACCCCAGCACAAGAACTACA	RT-qPCR	this study

Target	Sequence (5′-3′) f and r	Application	Reference
ETC chr9.2<br>chr9:44249309+44249402	ATTCCAGAGACCTTGGCTCG<br>GACTAGCAGCCTGCAACACC	RT-qPCR	this study
ETC chr7<br>chr7:20386293+20386436	CTGACCACTAGAGGGCACAC<br>TGAAGGTGTGAGCAAGGGTT	RT-qPCR	this study
ETC chr3<br>chr3:138189362+138189464	GACCCGGTTTAACGACTCCT<br>ACGTGGACGGAAATAGCCTAC	RT-qPCR	this study
IAPez	GCTCCTGAAGATGTAAGCAATAAAG<br>CTTCCTTGCGCCAGTCCCGAG	ChIP-qPCR	[11]
Intergenic	CAGCATTCCAGGAGGTTAGC<br>GTGCCTCATGTGCAGTCAGT	ChIP-qPCR	[3]

References

1. Rowbotham, S.P.; Barki, L.; Neves-Costa, A.; Santos, F.; Dean, W.; Hawkes, N.; Choudhary, P.; Will, W.R.; Webster, J.; Oxley, D.; et al. Maintenance of silent chromatin through replication requires SWI/SNF-like chromatin remodeler SMARCAD1. *Mol. Cell* **2011**, *42*, 285–296, doi:10.1016/j.molcel.2011.02.036.

2. Carrière, L.; Graziani, S.; Alibert, O.; Ghavi-Helm, Y.; Boussouar, F.; Humbertclaude, H.; Jounier, S.; Aude, J.-C.; Keime, C.; Murvai, J.; et al. Genomic binding of Pol III transcription machinery and relationship with TFIIS transcription factor distribution in mouse embryonic stem cells. *Nucleic Acids Res.* **2012**, *40*, 270–283, doi:10.1093/nar/gkr737.

3. Sachs, P.; Ding, D.; Bergmaier, P.; Lamp, B.; Schlagheck, C.; Finkernagel, F.; Nist, A.; Stiewe, T.; Mermoud, J.E. SMARCAD1 ATPase activity is required to silence endogenous retroviruses in embryonic stem cells. *Nat. Commun.* **2019**, *10*, 1335, doi:10.1038/s41467-019-09078-0.

4. Tucker, J.M.; Schaller, A.M.; Willis, I.; Glaunsinger, B.A. Alteration of the Premature tRNA Landscape by Gammaherpesvirus Infection. *mBio* **2020**, *11*, doi:10.1128/mBio.02664-20.

5. Winter, A.G.; Sourvinos, G.; Allison, S.J.; Tosh, K.; Scott, P.H.; Spandidos, D.A.; White, R.J. RNA polymerase III transcription factor TFIIIC2 is overexpressed in ovarian tumors. *Proc. Natl. Acad. Sci. U. S. A.* **2000**, *97*, 12619–12624, doi:10.1073/pnas.230224097.

6. Wossidlo, M.; Nakamura, T.; Lepikhov, K.; Marques, C.J.; Zakhartchenko, V.; Boiani, M.; Arand, J.; Nakano, T.; Reik, W.; Walter, J. 5-Hydroxymethylcytosine in the mammalian zygote is linked with epigenetic reprogramming. *Nat. Commun.* **2011**, *2*, 241, doi:10.1038/ncomms1240.

7. Rowe, H.M.; Jakobsson, J.; Mesnard, D.; Rougemont, J.; Reynard, S.; Aktas, T.; Maillard, P.V.; Layard-Liesching, H.; Verp, S.; Marquis, J.; et al. KAP1 controls endogenous retroviruses in embryonic stem cells. *Nature* **2010**, *463*, 237–240, doi:10.1038/nature08674.

8. Ding, D.; Bergmaier, P.; Sachs, P.; Klangwart, M.; Rückert, T.; Bartels, N.; Demmers, J.; Dekker, M.; Poot, R.A.; Mermoud, J.E. The CUE1 domain of the SNF2-like chromatin remodeler SMARCAD1 mediates its association with KRAB-associated protein 1 (KAP1) and KAP1 target genes. *J. Biol. Chem.* **2018**, *293*, 2711–2724, doi:10.1074/jbc.RA117.000959.

9. Zhang, Q.; Zhong, Q.; Evans, A.G.; Levy, D.; Zhong, S. Phosphorylation of histone H3 serine 28 modulates RNA polymerase III-dependent transcription. *Oncogene* **2011**, *30*, 3943–3952, doi:10.1038/onc.2011.105.

10. Torres-Adorno, A.M.; Lee, J.; Kogawa, T.; Ordentlich, P.; Tripathy, D.; Lim, B.; Ueno, N.T. Histone deacetylase inhibitor enhances the efficacy of MEK inhibitor through NOXA-mediated MCL1 degradation in triple-negative and inflammatory breast cancer. *Clin. Cancer Res.* **2017**, *23*, 4780–4792, doi:10.1158/1078-0432.CCR-16-2622.

11. Liu, S.; Brind'Amour, J.; Karimi, M.M.; Shirane, K.; Bogutz, A.; Lefebvre, L.; Sasaki, H.; Shinkai, Y.; Lorincz, M.C. Setdb1 is required for germline development and silencing of H3K9me3-marked endogenous retroviruses in primordial germ cells. *Genes Dev.* **2014**, *28*, 2041–2055, doi:10.1101/gad.244848.114.
